# Caring Flexibly? Working Conditions and the Supply of Adult Care

**DOI:** 10.1002/hec.70133

**Published:** 2026-07-16

**Authors:** Joan Costa‐Font, Wanying Wang

**Affiliations:** ^1^ Department of Health Policy London School of Economics and Political Science London UK

**Keywords:** flexible working, informal care, mental health, right to request flexible work, United Kingdom

## Abstract

Although flexible employment policies can help employed individuals balance caregiving and paid work, limited evidence has been devoted to examining the effect of working flexibly on the supply of care to older adults. In this paper, we study the impact of flexible working conditions on the supply of informal adult care and mental health. We exploit variation from the 2014 expansion of the *Right to Request Flexible Work* (RRFW) to employees in the UK. Our findings point to a gendered response to increased employment flexibility. We document a 1.3‐percentage‐point increase in the likelihood that men provide informal care within the household, alongside less regular daytime work, greater control over working hours, and higher engagement in home production. In contrast, among potential female caregivers, we find that the reform reduced the probability of high‐intensity caregiving, which is typically incompatible with employment or related activities. We document that the increased workplace flexibility not only encourages caregiving but also helps reduce gender disparities in unpaid care. We additionally find suggestive evidence of improved mental health outcomes, particularly among men.

## 1 Introduction

The rising of care needs, combined with limited public resources, makes supporting caregivers^1^ more important than ever. Nonetheless, a substantial share of adult care is still delivered by informal caregivers, often family members, who take on caregiving responsibilities out of necessity or due to social norms of care rather than choice. Many caregivers are expected to combine caregiving with paid employment, which creates a significant time burden that adds to the existing well‐being consequences of being a caregiver. Indeed, across different settings, evidence shows that caregiving is associated with reduced employment productivity (Keita Fakeye et al. 2023), more frequent work interruptions, and adverse effects on mental health (Urwin, Lau, Grande, and Sutton 2023a; Chang et al. 2010).

As work and care compete for time, a common response among caregivers is to switch to less demanding jobs, reduce labour supply intensity when possible, or exit the labour market altogether (Arrieta and Li 2023), including through early retirement (Costa‐Font and Vilaplana‐Prieto 2023). These adjustments often entail reductions in working hours that can lead to financial stress, career stagnation, and loss of professional identity (Akyol and Nolan 2025; Mozhaeva 2021; Spann et al. 2020; Longacre et al. 2016), which are all determinants of poorer mental health. Caregiving also imposes emotional (Costa‐Font et al. 2023), financial (Spann et al. 2020), and health‐related burdens (Carrino et al. 2023; Schmitz and Stroka 2013). However, the caregiving burden is far from being evenly distributed. Female caregivers disproportionately shoulder more caregiving responsibilities, which contributes to persistent gender inequalities in employment and earnings (Maestas et al. 2024), as well as worse mental health (Madia et al. 2023).

Such caregiving challenges are especially pronounced in countries where access to subsidised caregiver support is subject to strict means testing. Adult caregivers, however, are far from a residual minority. Approximately one in seven employees combines paid work with caregiving responsibilities, and around 8% of the population provides informal care in the United Kingdom (UK) (Carers 2019). Tensions between work and care have intensified over time, with evidence suggesting that caregiving contributes to workforce attrition in the UK, particularly in the absence of flexible work arrangements (Gonzales et al. 2015; Schneider et al. 2013). Hence, it is fundamental to identify cost‐effective policy interventions that allow caregivers to remain in the labour market while meeting care demands. One such intervention is the expansion of flexible working arrangements (FWAs), which expanded eligibility to request flexible work from a selective group (mainly parents of young children) to all employees, allowing greater control over when, where, or how work is performed. Increased access to FWAs may ease time constraints by enabling caregivers to adjust schedules, leave work earlier without reducing total hours, or better coordinate paid work with caregiving duties. It could also improve mental health and alleviate strain by increasing autonomy and reducing the psychological burden associated with balancing competing responsibilities. Mental health is hence shaped not only by working conditions, but also by the tasks and responsibilities individuals face both within and outside the workplace.

This paper examines the extent to which the increased exposure to FWAs affects informal caregiving provision and mental health among working‐age adults. We exploit the expansion of flexible working rights in the UK following the implementation of the Children and Families Act 2014, known as the Right to Request Flexible Work (RRFW). Using data from the UK Household Longitudinal Study (UKHLS), a nationally representative panel survey with rich information on employment, health, and family dynamics, our empirical strategy compares individuals who lacked access to flexible working arrangements prior to the reform (the treated group) with those who already had access (the control group). To improve the comparability between groups and enhance causal interpretation, we implement Propensity Score Matching, followed by an event study specification within a Difference‐in‐Differences framework to estimate the causal effects of the RRFW.

We first show that the RRFW expansion significantly increased, as expected, both the availability and uptake of flexible working arrangements. Given the strongly gendered nature of informal caregiving, we further examine the heterogeneous effects by gender and caregiving status. Our findings suggest that among men, the reform increased their likelihood of providing informal care within the household by 1.3 percentage points (a 28.7% increase relative to the pre‐reform mean), and by 4.9 percentage points among those who are potential caregivers.^2^ In contrast, among female employees who are also potential carers, we find a reduction in the provision of highly intensive caregiving exceeding 50 hours per week.

The estimated effects on mental health generally point towards improvements, particularly among men, although many of the estimates are not statistically significant. Importantly, the limited overall changes in mental health outcomes should not necessarily be interpreted as evidence of no mental health benefits from flexible working arrangements. Rather, the findings are consistent with the possibility that increased workplace flexibility helped offset some of the negative psychological consequences associated with caregiving. In other words, flexible working may have enabled individuals to absorb additional caregiving demands without experiencing corresponding deterioration in mental health.

To shed light on potential mechanisms, we examine changes in time use, work schedules, and household task allocation. We find that increased employment flexibility is associated with shifts in work schedules among men, including less regular daytime work and greater autonomy over working hours, which likely facilitated their increased caregiving involvement and improved mental health. We also observe an improved autonomy over working hours among women, and find evidence of improved coordination between partners, suggesting an impact of employment flexibility on the reallocation of domestic responsibilities.

This paper contributes to several strands of the literature. First, it adds to research on the gendered nature of informal caregiving and the role of workplace flexibility in shaping caregiving responsibilities. A large body of work shows that informal care is highly gendered, with women disproportionately bearing unpaid caregiving responsibilities (Carmichael and Charles 2003; Hiedemann and Stern 1999), while men often enter caregiving only after substantial labour supply disruptions (Maestas et al. 2024). Recent evidence suggests that flexible working arrangements may help alter these dynamics. Studies using data from the COVID‐19 period in Japan show that working from home increases men's time spent on household work and family activities (Inoue et al. 2024). Related evidence from childcare policies and parental working from home policies increases fathers' involvement in care and housework, reducing gender gaps in unpaid work (Tamm 2019; Patnaik 2019; Schüller 2026). Other studies find that relaxing work constraints, such as through paid sick leave, increases caregiving (Arora and Wolf 2024), and that flexible work is associated with better general health and mental health outcomes (Spearing 2025; Agnoletto 2024; Shifrin and Michel 2022; Chandola et al. 2019). However, the extent to which job flexibility promotes gender equality in informal adult caregiving and its implications for caregivers' mental health remain underexplored. This paper addresses this gap by examining how increased exposure to flexibility through a regulatory expansion affects caregiving and mental health outcomes across genders.

Second, we contribute to the literature on work–care trade‐offs faced by caregivers. Existing research shows that workplace inflexibility is a key driver of labour force exit among caregivers (Koreshi and Alpass 2023; Bainbridge and Townsend 2020). Evidence from New Zealand indicates that caregivers are more likely than non‐caregivers to use flexible working arrangements (Koreshi and Alpass 2023), while greater availability of flexibility improves perceptions of employer support (Bainbridge and Townsend 2020). Our study builds on this work by testing whether increased access to flexibility causally reduces the tension between paid work and informal caregiving.

Finally, this paper contributes to the literature on flexible working policies in the UK. Xue et al. (2026) study the 2014 RRFW reform in the general population and its effects on health and well‐being using earlier eligibility rules, though their findings are constrained by limited adoption prior to national rollout. Our analysis instead focuses on a more clearly treated group of employees who previously lacked access to flexible working arrangements, uses a more robust causal specification, and explicitly examines informal adult caregiving. Earlier work on the 2003 UK Flexible Working Act documented increased uptake of flexible work among mothers but no clear health effects (Avendano and Panico 2018). While other studies find positive associations between access to flexible work and caregiving hours (Bryan 2012), the evidence has not established causal links. We extend this literature by providing evidence on the effects of increased exposure to flexible work induced by the RRFW among workers, and by examining the mechanisms through which flexibility affects caregiving behaviour and mental health.

The remainder of the paper is organised as follows. Section 2 outlines the policy context and conceptual framework. Section 3 describes the data and empirical strategy. Section 4 presents the main results. Section 5 reports robustness checks. Section 6 concludes and discusses the implications.

## 2 Background

### 2.1 Flexible Working Arrangements

Flexible working arrangements, which allow employees to adjust their working hours, schedules, or location, have increasingly been recognised as an important policy intervention for improving work–life balance and supporting individuals with caregiving and other non‐work responsibilities. In the UK, flexible working has evolved from a targeted policy aimed at specific groups to a universal employment right available to all eligible employees.

The Right to Request Flexible Working (RRFW) was first introduced under the Employment Act 2002. The initial legislation established a statutory right for parents of young or disabled children to request changes to their working arrangements. Eligibility was limited to parents of children under six, or under 18 in the case of disability. Subsequent reforms modestly expanded eligibility. The Work and Families Act 2006 extended access to employees providing care for co‐residing dependent adults, and in 2009 the policy was broadened to include parents of children up to age 16. Despite these expansions, access to RRFW remained highly restrictive. Eligibility criteria excluded a substantial share of workers, particularly those involved in adult caregiving rather than childcare, and could also deter individuals who had not yet started providing care but lived with someone in need of support.

To date, the evidence on RRFW has not focused on caregivers. Moreover, empirical evidence indicates that both access to and uptake of flexible working arrangements remained uneven during this period. The uptake of RRFW was lower among men, and it was not available across the board, as many employees reported that flexible working was not available in their workplace. Structural barriers, gender norms, and workplace culture played a central role in limiting use (Atkinson and Hall 2009; Cook et al. 2021). In addition, it is worth mentioning that there is still a persistent stigma surrounding flexible working. For example, reinforced perceptions of lower productivity and weaker career commitment further discourage take‐up among eligible workers and contribute to gendered patterns of use (Chung 2020).

A major policy shift occurred with the Children and Families Act 2014, which transformed RRFW into a universal employment right by making the right extensive to anyone, including those providing any adult care (Cunningham et al. 2024). From 30 June 2014, any employee with at least 26 weeks of continuous service gained the right to formally request flexible work^3^.^4^ The reform also simplified procedural requirements, replacing detailed rules with a general duty for employers to handle requests in a reasonable manner. RRFW covers a wide range of flexible working arrangements, including job sharing, working from home, compressed hours, flexitime, and part‐time work. Estimates suggest that this reform expanded eligibility to more than 20 million workers.

### 2.2 Theoretical Mechanisms

The relationship between employment flexibility and informal caregiving can first be understood through theories of time allocation and time constraints, which are central to individual decision‐making. In the seminal model developed by Becker (1965), a fixed amount of time is allocated across paid work, unpaid household production (including caregiving), and leisure in order to maximise utility, subject to time and income constraints. Rigid working arrangements restrict when and how labour can be supplied, increasing the effective cost of combining paid work with caregiving responsibilities. As shown in labour supply models incorporating time costs and non‐convexities (Mincer 1962; Heckman 1974), such rigidities may discourage participation in unpaid care or lead individuals to reduce labour supply or exit employment when care needs arise. Hence, interventions that ease time constraints by expanding access to flexible working, such as RRFW, allow workers to adjust the timing, duration, or location of work without severing the employment relationship. We focus on and test in the later analysis three main mechanisms under this time constraint theoretical framework through which flexible working may affect informal care provision.

The first mechanism operates through changes in time allocation and time use. By lowering the fixed costs associated with reallocating time between paid work and unpaid care, flexible working arrangements may increase the feasibility of providing care while remaining employed. For example, reduced commuting time, compressed schedules, or shorter working hours may free up time that can be reallocated toward caregiving activities. Flexible work may therefore reduce the trade‐off between employment and care provision, particularly for workers facing substantial time constraints. The second mechanism operates through improved temporal coordination rather than changes in total time use. Even when total hours of paid work and leisure remain unchanged, greater control over the timing and location of work can reduce scheduling conflicts between employment and caregiving tasks. Flexibility in start and finish times or remote working arrangements may allow caregivers to respond to time‐specific care needs, such as accompanying relatives to medical appointments or providing supervision during particular periods of the day. In this context, flexible working may improve the compatibility between paid work and caregiving even in the absence of large changes in labour supply. The third mechanism relates to intra‐household allocation and bargaining. Evidence suggests that access to flexible working arrangements affects the coordination of work schedules within households (Bryan and Sevilla 2017). When caregiving responsibilities are shared among household members, increased access to flexibility may shift caregiving toward the individual with greater control over working time. An intra‐household perspective is therefore central to understanding both caregiving reallocation and associated mental health outcomes. Expanded access to flexible working may reduce barriers to caregiving among men, who on average face greater constraints from traditional full‐time work patterns and lower baseline involvement in unpaid care. For women, however, flexibility may have more ambiguous effects. On the one hand, it may reinforce existing gendered caregiving roles by making caregiving easier to combine with employment. On the other hand, greater flexibility among both partners may facilitate a more equal sharing of caregiving responsibilities, potentially reducing caregiving intensity among women.

The relationship between workplace flexibility and mental health is commonly understood through occupational stress and work‐family conflict frameworks. The theory of the Job Demand‐Control model (Karasek 1979) argues that psychological strain arises when workers face high job demands combined with low control over their work environment and schedule. These pressures may be further intensified when workers simultaneously face substantial caregiving responsibilities outside the workplace. Relatedly, the Job Demands‐Resources framework (Bakker and Demerouti 2007) expands on the previous explanation understanding and suggests that the effect of workplace demands on mental health depends on the availability of protective resources that help individuals cope with stress and role pressures. Another major framework is Work‐Family Conflict Theory (Greenhaus and Beutell 1985), which argues that conflict emerges when the demands of work and family roles become incompatible. In these settings, greater flexibility may improve mental health by increasing workers' control over the timing, location, and organisation of work, and address care needs that are often intensive, uncertain, and difficult to schedule in advance, thereby reducing the strain associated with balancing competing demands.

Although the broader relationship between working conditions and mental health is well established, the interaction between workplace flexibility, caregiving, and mental health remains less well understood. Importantly, the mental health effects of flexible working for caregivers are theoretically ambiguous. On the one hand, increased flexibility may mitigate stress and emotional strain. This may be particularly important in settings where long‐term care systems rely heavily on informal care provision and where formal social care resources remain limited. On the other hand, greater flexibility may also increase the total caregiving burden borne by workers if flexibility enables individuals to take on additional caregiving responsibilities rather than reducing existing pressures. Despite the increasing policy relevance of flexible working arrangements, empirical evidence on these mechanisms remains limited and has focused predominantly on childcare responsibilities and household labour coordination rather than adult caregiving.

## 3 Data and Empirical Strategy

### 3.1 Data

We use data from the UK Household Longitudinal Study (UKHLS), also known as Understanding Society (University of Essex Institute for Social and Economic Research 2023). UKHLS is a nationally representative longitudinal survey of individuals living in private households in England, Scotland, Wales, and Northern Ireland. It collects detailed information on demographic characteristics, education, labour market outcomes, health and mental health, family composition, caregiving activities, income, and civic participation. The first wave, conducted in 2009/10, surveyed around 40,000 households across the UK and provides rich information at both the individual and household levels. All original sample members aged 16 and over, as well as new household members who join sampled households over time, are followed and interviewed annually. The longitudinal structure allows individuals to be observed repeatedly, making the data well‐suited for analysing within‐individual behavioural responses to policy changes.

We construct the analytical sample using data from Waves 1 to 9 of UKHLS, covering the period before and after the 2014 expansion of the RRFW. Waves 10 onwards are excluded to avoid confounding effects associated with the COVID‐19 pandemic. The primary sample consists of individuals aged 16 and over who are observed in paid employment throughout the study period. Focusing on continuously employed individuals allows us to study changes in caregiving without conflating them with transitions into or out of employment. Given that the policy is particularly relevant for workers with potential caregiving responsibilities, we also construct a secondary sample of potential caregivers. This group is defined as employees who report having at least one family member^5^ with a disability or long‐term health condition requiring care in Wave 4 (2012/13), prior to the policy implementation.

### 3.2 Empirical Strategy

#### 3.2.1 Identification Strategy

We exploit the 2014 expansion of the RRFW as a source of variation in access to flexible working arrangements. The reform extended the right to request flexible working to all employees, thereby increasing exposure to flexibility among workers employed in workplaces that previously did not offer such arrangements. We estimate the impact of the increased exposure on informal caregiving and mental health using an event‐study framework combined with a Difference‐in‐Differences (DiD) design. The event study approach is a key complementary approach that allows us to test the core parallel trend assumption and to track the dynamic treatment effects over time. The strategy compares changes in outcomes over time between individuals who gained access to flexible working following the reform (treated group) and those who already had access prior to the reform (control group), following Avendano and Panico (2018). Treatment status is defined based on self‐reported access to flexible working arrangements at the workplace. Respondents are asked whether any flexible working arrangements are available to them. Individuals who report no access to any form of flexible working prior to the reform are classified as treated, while those who report access to at least one arrangement before the reform form the control group.

The identifying assumption of the DiD design is that, absent the RRFW expansion, outcomes for treated and control individuals would have followed parallel trends. The event study specification allows this assumption to be assessed empirically by estimating dynamic treatment effects in the pre‐reform period and checking whether such effects are not different from zero. The absence of statistically significant pre‐trends provides support for the validity of the design, while the post‐reform coefficients trace the evolution of policy effects over time. However, because access to flexible working is not randomly assigned, treated and control individuals may differ systematically in characteristics that also influence caregiving behaviour, potentially threatening the plausibility of parallel trends.

To address the imbalance in observed pre‐treatment characteristics, we implement Propensity Score Matching (PSM) prior to estimation. The propensity score is estimated using a logistic regression of treatment status on baseline covariates measured before the reform, including age, gender, marital status, educational attainment, National Statistics Socio‐economic Classification (NS‐SEC), and Standard Industrial Classification (SIC). These characteristics are strong predictors of both access to workplace flexibility and informal caregiving behaviour. We apply nearest‐neighbour matching with replacement, and the resulting matched sample is used in the main DiD and event study analyses. To further reduce residual confounding, all baseline covariates used for matching are also included as controls in the regression models.

It is worth noting that informal caregiving tends to be highly persistent over time, with pre‐treatment caregiving status strongly predicting post‐treatment outcomes. To account for this, we examine heterogeneity by baseline caregiving status through subgroup analyses. This approach allows us to assess whether increased workplace flexibility encourages non‐caregivers to begin providing care, and whether it helps existing caregivers maintain their caregiving role or adjust the intensity of care they provide. We do not rely on matching on prior caregiving status, as doing so would condition on an outcome closely related to our margin of interest and would mechanically limit post‐reform transitions into caregiving, potentially underestimating the effects on the extensive margin.

**FIGURE 1:**
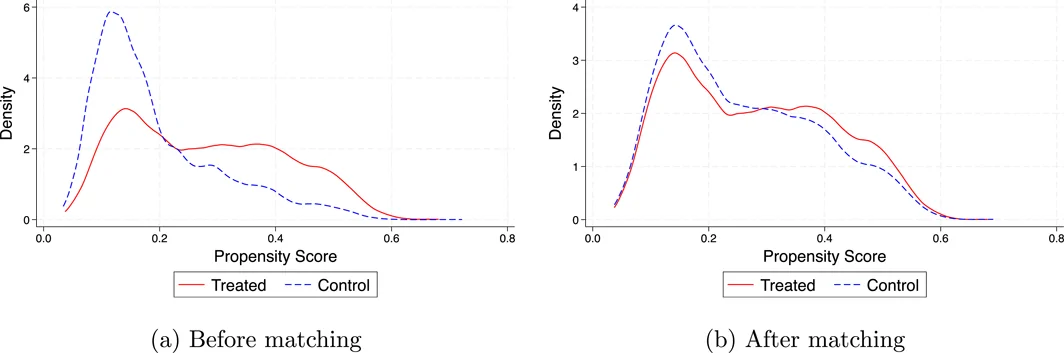
Propensity score distributions. The Figure shows the propensity score distributions for treated and control groups before (a) and after (b) matching. The data are drawn from the UK Household Longitudinal Study.

**FIGURE 2:**
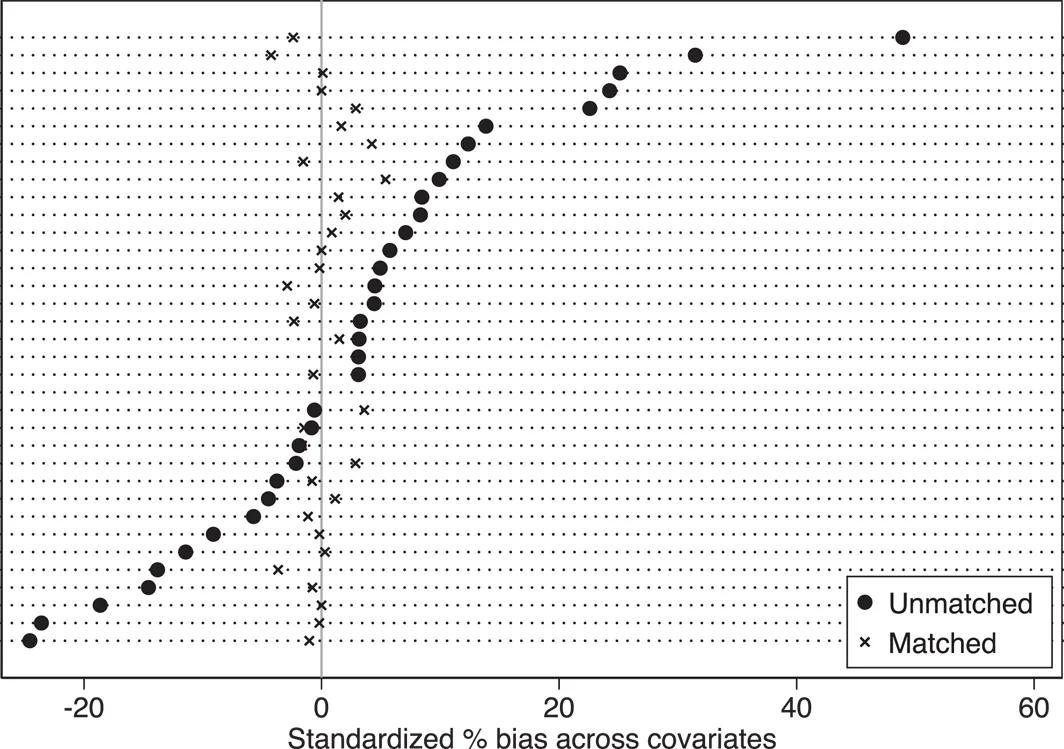
Standardised covariate imbalance before and after matching. The Figure shows the standardised percentage differences in key covariates between treated and control groups for the unmatched and matched samples. Standardised differences are computed as the mean difference between groups, divided by the pooled standard deviation, and expressed as a percentage.

**FIGURE A1:**
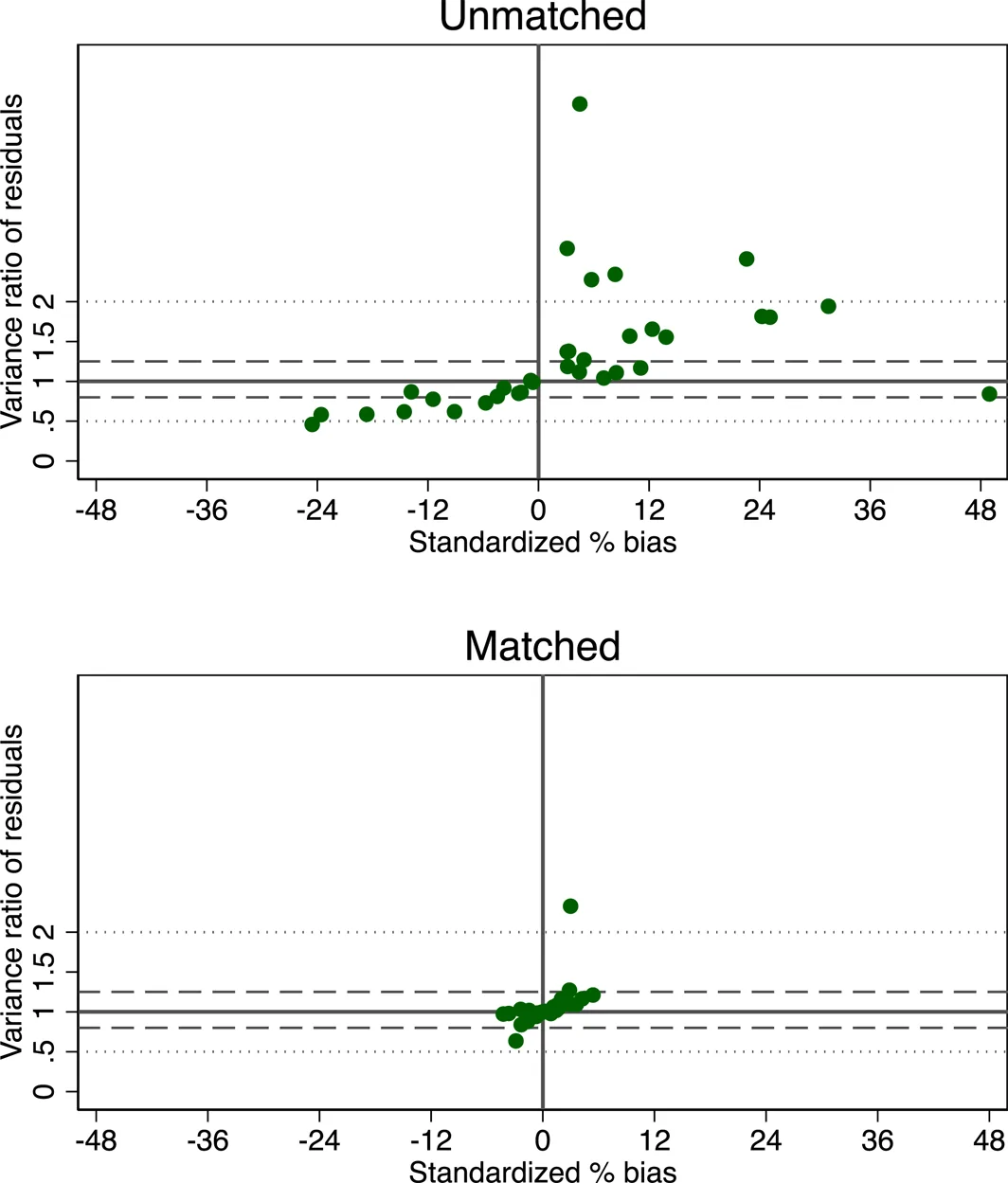
Covariate balance for unmatched and matched samples. The Figure compares covariate balance for the unmatched and matched samples using standardised percentage bias and variance ratios. Standardised bias measures the percentage difference in means between the treated and control groups, while the variance ratio assesses distributional similarity. The dashed lines indicate common thresholds for accepTable balance. The data are from the UK Household Longitudinal Study.

The final primary sample includes 5048 individuals observed across Waves 1 to 9, while the secondary sample of potential caregivers consists of 1205 individuals. Table A1 in the Appendix reports descriptive statistics for treated and control groups before and after matching. Prior to matching, substantial differences exist between the groups. The treated group includes a higher share of men and is more concentrated in semi‐routine occupations and in industries such as manufacturing, construction, and accommodation and food services, reflecting well‐documented sectoral disparities in access to flexible working arrangements (Belloni et al. 2022). Age and educational attainment also differ, while marital status distributions are more similar. The matching procedure substantially improves balance across observed characteristics. Figures 1 and 2 show the distribution of propensity scores and covariate balance before and after matching, and Figure A1 in the Appendix summarises reductions in standardised biases across all covariates.

#### 3.2.2 Variables of Interest

Table 1 presents the key summary statistics for the matched primary sample of continuously employed individuals. The variables describing the workplace flexibility include the number of flexible working arrangements available, the use of any flexible working arrangement, and the use of informal flexible working arrangements. On average, individuals in the control group report greater availability and use of flexible working arrangements.^6^

**TABLE 1:** Summary statistics of key variables (matched sample).

Variables	Treated				Control			
	*N*	Mean	Min	Max	*N*	Mean	Min	Max
Number of FWAs available	9202	0.522	0	7	5867	1.804	0	7
Use any FWAs	9202	0.129	0	1	5867	0.450	0	1
Use informal FWAs	9280	0.503	0	1	5936	0.677	0	1
Care within household	18,141	0.042	0	1	12,847	0.058	0	1
Care outside household	19,965	0.106	0	1	12,974	0.119	0	1
Care > 20 hours	17,948	0.020	0	1	11,713	0.027	0	1
Care > 50 hours	17,948	0.005	0	1	11,713	0.008	0	1
SF‐12 MCS	18,766	50.12	1.990	75.54	12,358	49.94	3.180	72.87
GHQ likert scale (reversed)	18,675	25.42	0	36	12,301	25.31	0	36
Work hours	20,184	38.07	0.100	97	14,064	35.06	0.100	97.90
Overtime hours	19,633	3.976	0	75	12,753	3.895	0	95
Commuting hours	19,255	0.423	0	16.62	12,403	0.433	0	5
Housework hours	9764	7.401	0	70	6398	8.306	0	50
Weekend work	9374	0.598	0	1	6035	0.547	0	1
Regular daytime work	9343	0.672	0	1	6026	0.678	0	1
Working‐hour autonomy	9363	0.343	0	1	6028	0.501	0	1
Working‐manner autonomy	9363	0.812	0	1	6024	0.853	0	1
Within‐couple: Mainly cooking	7025	0.271	0	1	4447	0.305	0	1
Within‐couple: Mainly grocery shopping	7025	0.246	0	1	4446	0.297	0	1
Within‐couple: Mainly cleaning	7025	0.246	0	1	4447	0.282	0	1
Within‐couple: Mainly washing	7024	0.249	0	1	4447	0.316	0	1
Within‐couple: Mainly gardening	7012	0.396	0	1	4438	0.365	0	1

*Note:* The Table presents summary statistics for key variables using the matched sample. The data are from Waves 1 to Wave 9 of the UK Household Longitudinal Study. Summary statistics are reported separately for the treated and control groups.

The main caregiving outcomes capture both the incidence and intensity of informal care provision. UKHLS collects annual information on care provided within and outside the household, as well as weekly hours spent on caregiving. Weekly hours are reported in categorical bands ranging from 0 to 4 to 100 or more hours. Using this information, we construct measures at both the extensive and intensive margins. At the extensive margin, we examine whether the individual provides any informal care within or outside the household. At the intensive margin, we define indicators for high‐intensity caregiving based on thresholds of more than 20 hours and more than 50 hours per week. These thresholds are commonly used in the literature and policy contexts (Pennington et al. 2025; Urwin, Lau, Grande, and Sutton 2023b; Carmichael et al. 2010). Providing care for more than 20 hours per week indicates regular caregiving that can potentially interfere with standard full‐time work schedules, while care exceeding 50 hours per week is interpreted as a proxy of full‐time caregiving, often linked to reduced labour supply. On average, individuals in the treated group are slightly less likely to provide informal care and less likely to engage in intensive caregiving, reflecting their more limited access to workplace flexibility before the reform.

To examine mental health outcomes, we use two complementary measures that are widely employed in the health economics and public health literature (Martínez‐Jiménez 2023; Arulsamy and Delaney 2022): the Mental Component Scale of the 12‐item Short‐Form Health Survey (SF‐12 MCS) and the General Health Questionnaire Likert scale (GHQ). The two measures capture related but different dimensions of mental health, as the GHQ primarily measures short‐term psychological distress, whereas the SF‐12 MCS captures broader mental health‐related quality of life based on population‐normed functioning measures.

The SF‐12 MCS is a continuous index ranging from 0 to 100, with higher values indicating better mental health functioning. The measure captures four broad domains of mental well‐being. The mental health domain assesses feelings of calmness, peacefulness, nervousness, and depression. The role‐emotional domain evaluates the extent to which emotional problems interfere with work and other daily activities. The social functioning domain measures the extent to which physical or emotional health interferes with normal social activities. Finally, the vitality domain captures energy levels and fatigue. The GHQ Likert scale is a widely used measure of psychological distress ranging from 0 to 36. To facilitate consistent interpretation across mental health outcomes, we reverse‐code the GHQ Likert scale so that higher values indicate better mental health, as with SF‐12 MCS. Respondents report how their recent psychological state differs from their usual condition. The GHQ consists of 12 items covering concentration, sleep quality, sense of purpose, decision‐making ability, stress, difficulty overcoming problems, enjoyment of daily activities, coping ability, feelings of depression, confidence, self‐worth, and overall happiness. On average, individuals in the control group report slightly lower levels of mental health across both mental health measures.

Finally, we construct a set of potential mediating variables based on the theoretical frameworks to explore mechanisms linking workplace flexibility to caregiving and mental health outcomes. The possible channels include changes in time allocation, temporal coordination of work and caregiving responsibilities, and the intra‐household distribution of tasks. To examine these channels, we include continuous measures of time use, including weekly working hours, overtime hours, commuting time, and hours spent on housework. We also examine work schedule characteristics through indicators for weekend work, regular daytime schedules, and reported autonomy over working hours and work organisation. To capture intra‐household allocation of domestic responsibilities, we further examine responsibility for household tasks, including cooking, grocery shopping, cleaning, washing, and gardening. These variables are coded as dichotomous indicators equal to one if the respondent reports being mainly responsible for the corresponding task. Descriptive statistics suggest that across the waves, individuals with limited access to flexible working arrangements tend to work longer hours, perform more overtime, experience lower levels of workplace autonomy, and are more likely to work weekends. Within households, they are also generally less likely to report primary responsibility for domestic tasks, with the exception of gardening.

**FIGURE A2:**
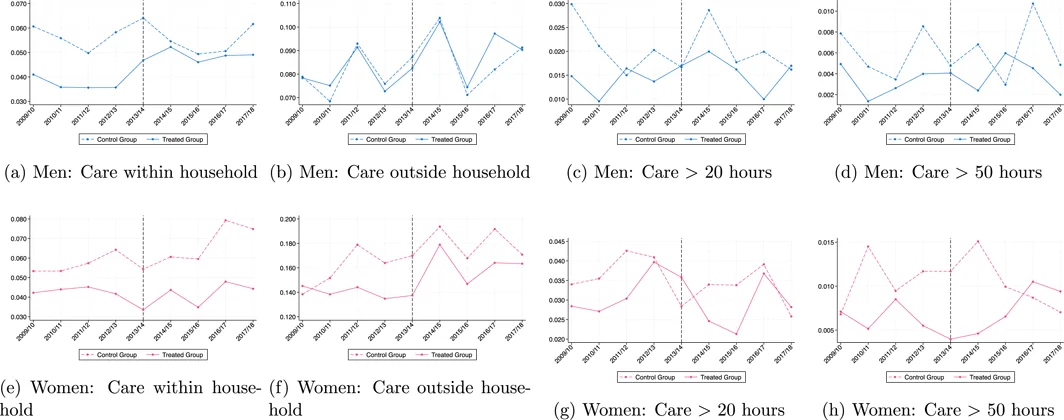
Descriptive trends in informal care provision by gender. The Figure shows mean shares of individuals providing informal care by year, separately for men and women. (a) and (e) report care provided within the household, (b) and (f) report care provided outside the household, (c) and (g) report intensive caregiving of more than 20 hours per week, and (d) and (h) report intensive caregiving of more than 50 hours per week. The vertical dashed line marks the policy implementation period. The analysis uses data from Waves 1 to Wave 9 of the UK Household Longitudinal Study.

**FIGURE A3:**
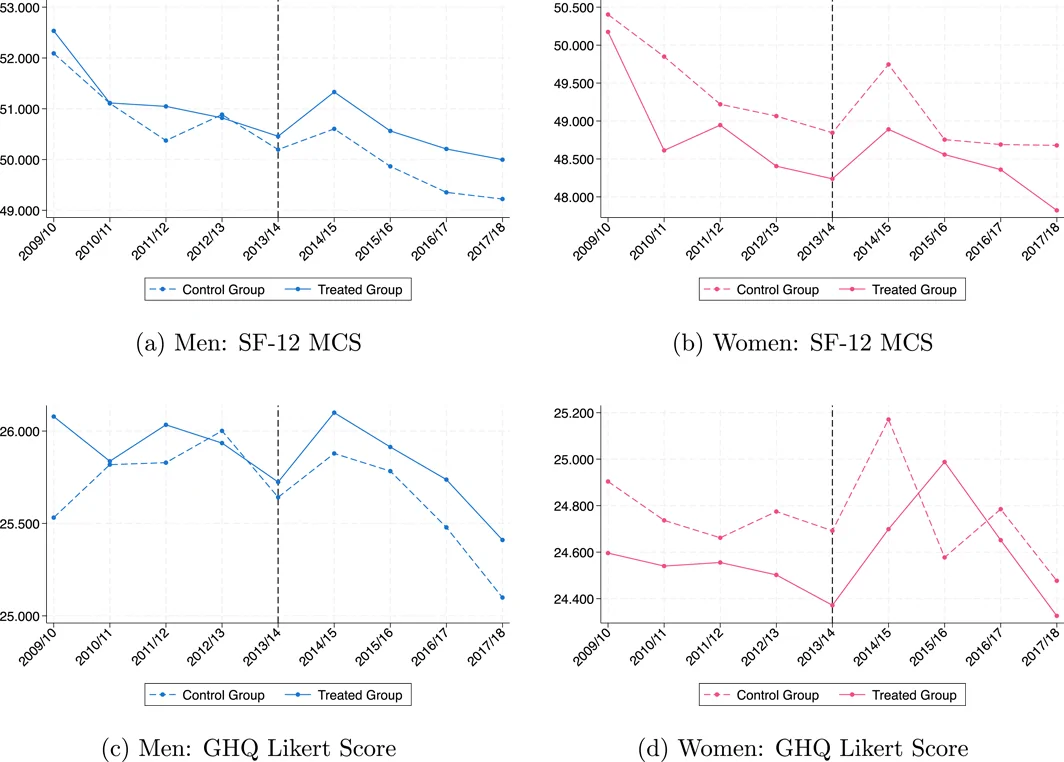
Trends in mental well‐being scores by gender. The Figure shows the mean of mental health indicators by year, separately for men and women. (a) and (b) report SF‐12 MCS, (c) and (d) report GHQ Likert score. The vertical dashed line marks the policy implementation period. The analysis uses data from Waves 1 to Wave 9 of the UK Household Longitudinal Study.

Figure A2 presents descriptive trends in caregiving outcomes for treated and control groups between 2009 and 2018. Among men, we observe a noticeable increase in within‐household caregiving among the treated group following the RRFW reform, bringing levels close to those of the control group. In contrast, caregiving trends among women change little for the treated group, while the control group shows a modest increase toward the end of the period. For care provided outside the household, trends for men are similar across groups, whereas treated women exhibit a slight increase relative to controls. Trends in intensive caregiving show greater volatility. Figure A3 shows descriptive trends in mental health. For both mental health measures and for both men and women, the scores display a gradual downward trend over time. GHQ scores for men show a larger decrease after the reform, despite still being higher than scores for women, while women's GHQ scores show greater post‐reform fluctuation, despite similar pre‐reform trends.

#### 3.2.3 Event Study Specification

We estimate the following event study specification to examine pre‐reform trends and post‐reform dynamics, following Gulyas et al. (2023) and Fadlon and Nielsen (2021):

(1) $$Y_{it}=\alpha_{i}+\lambda_{t}+\sum_{j=k}\gamma_{j}1\left[r_{it}=j\right]+\omega X^{\prime }+\epsilon_{it}$$

Here $Y_{it}$ corresponds to the outcome variables. $1\left[r_{it}=j\right]$ is an indicator of the time relative to the implementation of the regulation. It equals one when the respondent i in period t is j years away from the implementation of the flexible work regulation, where k∈{−4,−3,−2,0,1,2,3,4}. $r_{it}=-1$ stands for being surveyed before July 2014, which is the baseline time. The coefficient $\gamma_{j}$ captures dynamic treatment effects relative to the baseline. $\alpha_{i}$ and $\lambda_{t}$ are individual and time‐fixed effects. The vector $X^{\prime }$ includes baseline demographic and socioeconomic covariates, including age, marital status, education, NS‐SEC, SIC, household composition, health status, employment status, and employer size. We use the series of coefficients $\gamma_{j}$ to assess whether a significant difference is evident in outcome trends between the matched treated and control groups before the regulation.

#### 3.2.4 Difference‐In‐Differences Specification

The Difference‐in‐Differences specification is defined as follows:

(2) $$Y_{it}=\alpha_{i}+\lambda_{t}+\beta_{1}Treated_{i}\times Post_{it}+\omega X^{\prime }+\epsilon_{it}$$

In the equation, $Y_{it}$ denotes the set of outcome variables. $\alpha_{i}$ is the individual fixed effect, and $\lambda_{t}$ is the time fixed effect. $Treated_{i}\times Post_{it}$ equals one for treated individuals in the post‐reform period, and $\beta_{1}$ captures the average effect of exposure to the RRFW expansion. $X^{\prime }$ are the covariates, same as in the event study model. $\epsilon_{it}$ is the error term. Results are presented separately by gender, for the full sample, and for the secondary sample of potential caregivers. We further explore heterogeneity across subgroups, test hypothesised mechanisms, and assess robustness using alternative specifications.

## 4 Results

### 4.1 Changes in Availability and Use of FWAs

We begin by assessing whether the RRFW expansion led to changes in the availability and use of flexible working arrangements, using the DiD specification in Equation (2). Table 2 presents the estimated effects. We perform this first‐stage test to verify whether the reform led to real changes in workplace flexibility.

**TABLE 2:** Impact of RRFW on flexible work arrangements.

Variables	No. of FWAs		Use any FWAs		Use informal FWAs	
	Men	Women	Men	Women	Men	Women
	(1)	(2)	(3)	(4)	(5)	(6)
Total population						
Treated × post	0.528***	0.866***	0.133***	0.212***	0.065***	0.094***
	(0.055)	(0.070)	(0.017)	(0.023)	(0.019)	(0.026)
Controls	Yes	Yes	Yes	Yes	Yes	Yes
Observations	8942	5282	8942	5282	9043	5330
R‐squared	0.647	0.649	0.563	0.630	0.560	0.547
% of pre‐reform mean	71.50	76.95	81.31	63.47	11.51	17.84
Potential carers						
Treated × post	0.649***	0.812***	0.100***	0.216***	0.114***	0.079*
	(0.111)	(0.137)	(0.037)	(0.041)	(0.043)	(0.046)
Controls	Yes	Yes	Yes	Yes	Yes	Yes
Observations	1993	1414	1993	1414	2015	1433
R‐squared	0.648	0.646	0.587	0.678	0.567	0.607
% of pre‐reform mean	85.47	72.78	62.21	61.18	19.67	14.36

*Note:* The Table presents two‐way fixed effects estimates of the impact of the expansion of the Right to Request Flexible Work regulation on flexible work arrangements by gender. The data are from Waves 1 to Wave 9 of the UK Household Longitudinal Study. The upper panel reports results for the total population, while the lower panels provide subgroup estimates for potential caregivers. All specifications control for age and age squared, marital status, education, National Statistics Socio‐Economic Classification, Standard Industry Classification, number of parents in the household, co‐residence with a spouse, number of children, general health, full‐time employment status, and employer size. Individual and time fixed effects are included. Robust standard errors are clustered at the individual level.

^*^ *p* < 0.1.

**p < 0.05.

^***^ *p* < 0.01.

Across the full sample, we find, as expected, that an increased exposure to the RRFW reform significantly increases the number of FWAs available in the workplace for both men and women. The estimated increase is approximately 0.53 arrangements for men and 0.87 for women. Similarly, we find that actual use of FWAs is higher among women who respond more strongly to the reform than men. The probability of using any FWA rises by 13.3 percentage points for men and by 21.2 percentage points for women. The policy also gives rise to positive spillovers on informal flexibility. That is, the likelihood of using informal FWAs increases by 6.5 percentage points for men and 9.4 percentage points for women, suggesting that formal rights may facilitate broader cultural acceptance of flexible practices within workplaces.

Results for the subsample of potential caregivers are broadly similar. Both men and women experience increases in the number of available FWAs and in the likelihood of using formal and informal flexibility. One noTable difference is that, within this group, men exhibit a larger increase in the use of informal FWAs than women following the reform. The findings provide strong first‐stage evidence that the RRFW expansion meaningfully improved access to and use of flexible working arrangements.

### 4.2 Event Study Estimates

We present the event‐study estimates before the baseline DiD results, as the event‐study framework provides an important diagnostic test for the credibility of the research design by examining whether treated and control groups followed similar trends prior to the policy change. The absence of statistically or economically meaningful pre‐treatment differences supports the parallel trends assumption underlying the DiD identification strategy.

**FIGURE 3:**
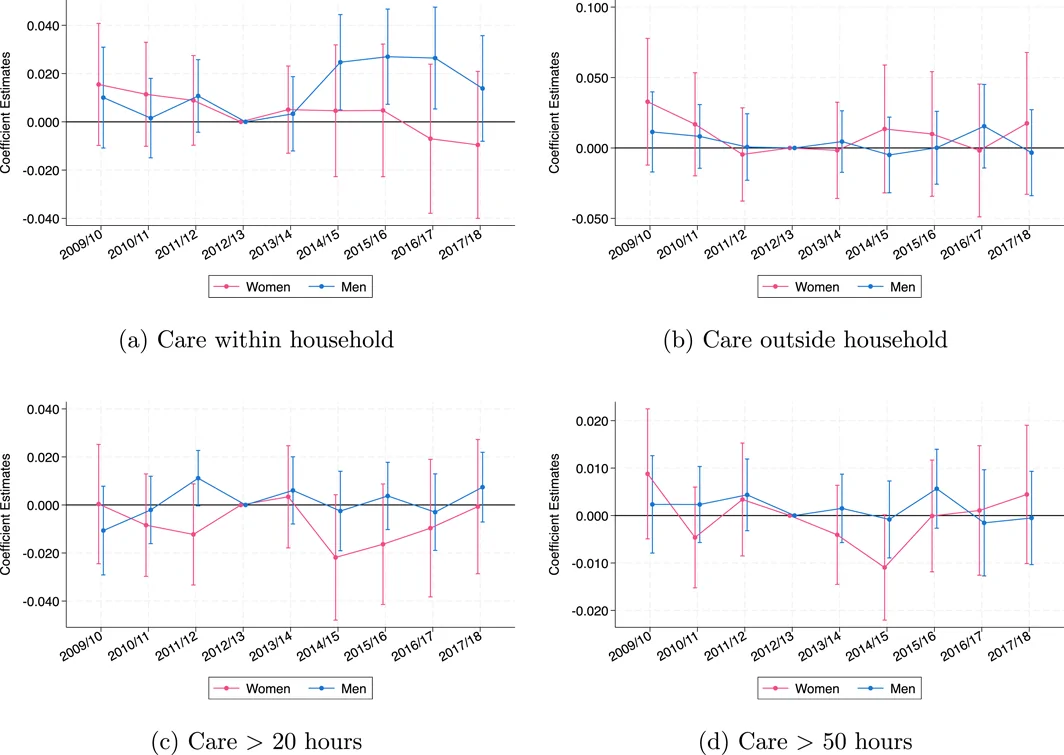
Event study estimates of the impact of RRFW on informal care provision. The Figure presents event study estimates of the impact of the Right to Request Flexible Work expansion on informal care provision. (a) shows coefficient estimates for providing care within the household, (b) shows estimates for providing care outside the household, (c) presents estimates for caring for more than 20 hours per week, while (d) presents estimates for caring for more than 50 hours per week. The analysis uses data from Waves 1 to Wave 9 of the UK Household Longitudinal Study, with Wave 4 (2012/2013) as the baseline. The vertical lines represent 95% confidence intervals around the coefficient estimates.

Figure 3 displays the event study estimates retrieved from Equation (1), which illustrate the dynamic effects of the RRFW expansion on informal caregiving outcomes vary by gender. For men, the estimates show a clear and persistent increase in the probability of providing informal care within the household following the 2014 reform. The increase emerges immediately after the policy introduction and remains statistically significant through 2017. In contrast, for women, there is little evidence of a corresponding increase in caregiving participation at the extensive margin. Turning to intensive caregiving, the estimates indicate a modest decline among women, particularly in the short period immediately following the reform (2014–2015), while effects for men are small and statistically insignificant. Table A3 in the Appendix reports the corresponding point estimates. For men, the increase in within‐household caregiving ranges between 2.8 and 3.1 percentage points across post‐reform years. For women, the probability of providing intensive care declines by around 1.1 percentage points. Notably, the pre‐reform coefficients are close to zero and statistically insignificant, lending support to the parallel trends assumption.

**FIGURE 4:**
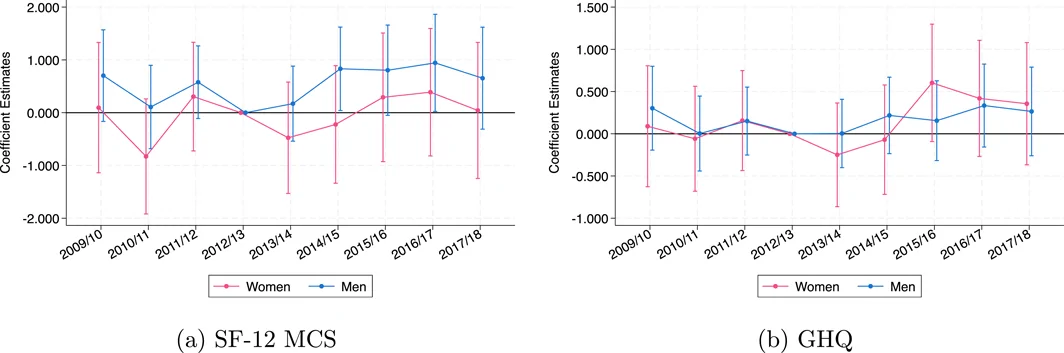
Event study estimates of the impact of RRFW on mental health. The Figure presents event study estimates of the impact of the Right to Request Flexible Work expansion on mental health. (a) shows coefficient estimates for the SF‐12 MCS, (b) shows estimates for the recoded GHQ Likert score. The analysis uses data from Waves 1 to Wave 9 of the UK Household Longitudinal Study, with Wave 4 (2012/2013) as the baseline. The vertical lines represent 95% confidence intervals around the coefficient estimates.

Figure 4 reports the event study estimates for mental health outcomes. For men, there is a suggestive improvement in mental health following the reform, reflected in a gradual increase in the SF‐12 MCS. Together with the observed increase in men's involvement in within‐household caregiving, this suggests that greater workplace flexibility enabled men to take on caregiving responsibilities and, at the same time, led to better mental health. For women, we observe a slight improvement in terms of GHQ measures post‐reform, though remain insignificant. As with caregiving outcomes, pre‐reform trends in both mental health measures appear parallel between treated and control groups.

**FIGURE A4:**
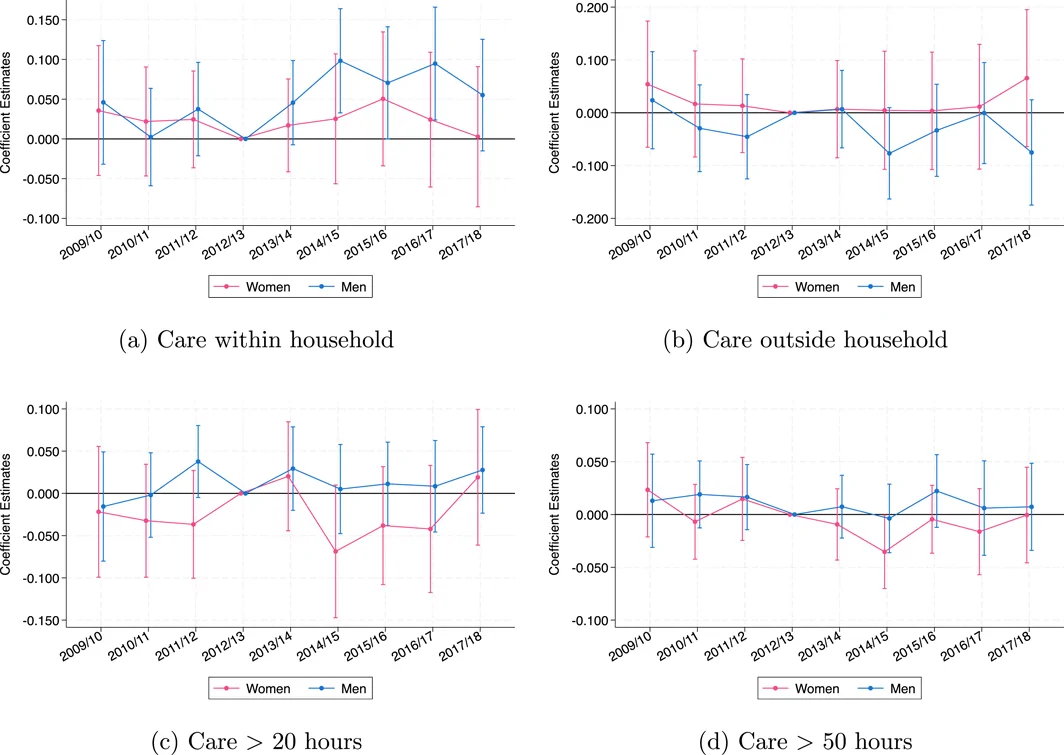
Event study estimates of the impact of RRFW on informal care provision among potential caregivers. The Figure presents event study estimates of the impact of the RRFW expansion on informal care provision among potential caregivers. (a) shows coefficient estimates for providing care within the household, (b) shows estimates for providing care outside the household, (c) shows estimates for caring for more than 20 hours per week, while (d) presents estimates for caring for more than 50 hours per week. The analysis uses data from Waves 1 to Wave 9 of the UK Household Longitudinal Study, with Wave 4 (2012/2013) as the baseline. The vertical lines represent 95% confidence intervals around the coefficient estimates.

**FIGURE A5:**
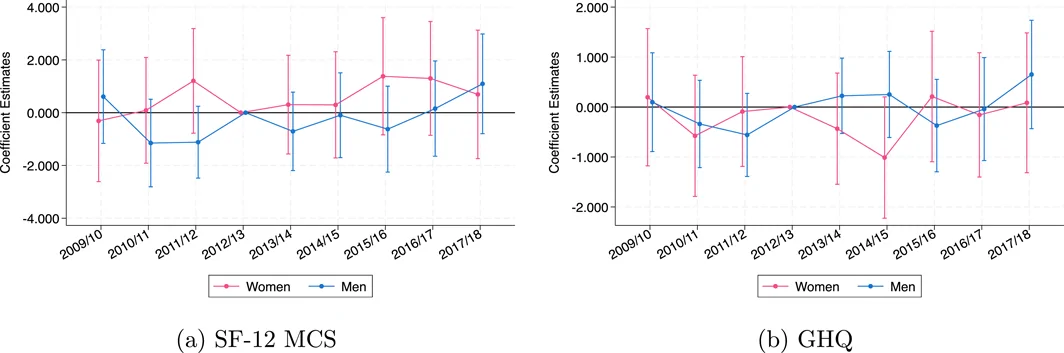
Event study estimates of the impact of RRFW on mental health among potential caregivers. The Figure presents event study estimates of the impact of the RRFW expansion on mental health among potential caregivers. (a) shows coefficient estimates for the SF‐12 MCS, (b) shows estimates for the GHQ Likert score. The analysis uses data from Waves 1 to Wave 9 of the UK Household Longitudinal Study, with Wave 4 (2012/2013) as the baseline. The vertical lines represent 95% confidence intervals around the coefficient estimates.

In the Appendix, Figures A4 and A5 restrict the analysis to the sample of potential caregivers, defined as individuals who reported at least one family member with a disability or long‐term health condition in the baseline wave. The event study estimates for caregiving outcomes in Figure A4 closely mirror the main findings but are larger in magnitude. Among men, the likelihood of providing within‐household care increases by around 10 percentage points in the year immediately following the RRFW expansion. Among women, we again observe a small reduction in intensive caregiving, measured both at the 20‐h and 50‐h thresholds. Figure A5 shows that effects on mental health for potential caregivers remain small and statistically insignificant for both genders.

### 4.3 Difference‐In‐Differences Results

#### 4.3.1 Impacts on Informal Care Provision

Conditional on the event‐study evidence supporting the validity of the design, we then present the standard DiD estimates to provide a summary of the average post‐treatment effect. Table 3 reports the DiD estimates for informal caregiving outcomes. The RRFW expansion increases the probability of men providing within‐household informal care by 1.3 percentage points, which is a 28.7% increase from the pre‐reform mean. In contrast, the effects on caregiving outside the household and on intensive caregiving are small and statistically insignificant. For women, the estimates point to a reduction in the supply of higher‐intensity care. The effects are larger among potential caregivers, which shows that men experience a 4.9 percentage point increase in the likelihood of providing within‐household care, while women experience a 1.9 percentage point reduction in the probability of providing more than 50 hours of care per week. We observe a clear gendered result. Men respond to the expansion of flexible working mainly at the extensive margin of caregiving, whereas women's response is concentrated in reductions in caregiving intensity.

**TABLE 3:** Impact of RRFW on informal care provision.

Variables	Care within HH		Care outside HH		Care > 20 hours		Care > 50 hours	
	Men	Women	Men	Women	Men	Women	Men	Women
	(1)	(2)	(3)	(4)	(5)	(6)	(7)	(8)
Total population								
Treated × post	0.013**	−0.007	−0.002	−0.002	0.001	−0.002	−0.001	−0.004
	(0.006)	(0.009)	(0.008)	(0.015)	(0.004)	(0.007)	(0.002)	(0.003)
Controls	Yes	Yes	Yes	Yes	Yes	Yes	Yes	Yes
Observations	17,536	10,380	19,875	11,722	17,740	10,602	17,740	10,602
R‐squared	0.616	0.596	0.467	0.527	0.493	0.475	0.385	0.448
% of pre‐reform mean	28.74	−14.81	−2.291	−1.501	7.622	−6.706	−27.65	−43.95
Potential carers								
Treated × post	0.049**	0.006	−0.011	−0.002	0.010	0.002	−0.004	−0.019**
	(0.020)	(0.026)	(0.025)	(0.039)	(0.013)	(0.021)	(0.010)	(0.009)
Controls	Yes	Yes	Yes	Yes	Yes	Yes	Yes	Yes
Observations	4200	2859	4444	3145	4299	3008	4299	3008
R‐squared	0.646	0.638	0.544	0.548	0.528	0.502	0.405	0.474
% of pre‐reform mean	29.69	3.884	−4.341	−0.591	16.57	1.960	−22.11	−72.68

*Note:* The Table presents two‐way fixed effects estimates of the impact of the expansion of the Right to Request Flexible Work regulation on informal care provision by gender. The data are from Waves 1 to Wave 9 of the UK Household Longitudinal Study. The upper panel reports results for the total population, while the lower panels provide subgroup estimates for potential caregivers. All specifications control for age and age squared, marital status, education, National Statistics Socio‐Economic Classification, Standard Industry Classification, number of parents in the household, co‐residence with a spouse, number of children, general health, full‐time employment status, and employer size. Individual and time fixed effects are included. Robust standard errors are clustered at the individual level.

*p < 0.1.

^**^ *p* < 0.05.

***p < 0.01.

#### 4.3.2 Impacts on Mental Health

Table 4 presents the estimated effects of the RRFW expansion on mental health. Consistent with the event study results, the estimates for the full sample indicate positive but statistically insignificant improvements in the SF‐12 Mental Component Score. Among the total population, the positive effects are larger for men than for women, while among potential carers, women exhibit larger improvements. The GHQ Likert estimates suggest generally positive effects of less psychological distress, but again, these effects are not statistically significant. Overall, the results suggest that the RRFW expansion led to improvements in mental health but with insignificant estimates.

**TABLE 4:** Impact of RRFW on mental health.

Variables	SF‐12 MCS		GHQ likert	
	Men	Women	Men	Women
	(1)	(2)	(3)	(4)
Total population				
Treated × post	0.285	0.010	0.122	0.147
	(0.223)	(0.328)	(0.121)	(0.186)
Controls	Yes	Yes	Yes	Yes
Observations	18,884	11,157	18,762	11,133
R‐squared	0.568	0.537	0.544	0.528
% of pre‐reform mean	0.557	0.0209	0.472	0.598
Potential carers				
Treated × post	0.376	0.467	0.350	−0.149
	(0.483)	(0.628)	(0.265)	(0.364)
Controls	Yes	Yes	Yes	Yes
Observations	4205	3005	4179	3000
R‐squared	0.599	0.555	0.567	0.523
% of pre‐reform mean	0.738	0.955	1.362	−0.614

*Note:* The Table presents two‐way fixed effects estimates of the impact of the expansion of the Right to Request Flexible Work regulation on mental health by gender. The data are from Waves 1 to Wave 9 of the UK Household Longitudinal Study. The upper panel reports results for the total population, while the lower panels provide subgroup estimates for potential caregivers. All specifications control for age and age squared, marital status, education, National Statistics Socio‐Economic Classification, Standard Industry Classification, number of parents in the household, co‐residence with a spouse, number of children, general health, full‐time employment status, and employer size. Individual and time fixed effects are included. Robust standard errors are clustered at the individual level.

*p < 0.1.

**p < 0.05.

***p < 0.01.

### 4.4 Heterogeneity Analysis

We next examine heterogeneity by caregiving status in the pre‐reform period. Specifically, we re‐estimate the main specifications separately for individuals who reported providing any informal care between 2009 and 2013 and those who did not. The results are reported in Table 5. Among men, the increase in within‐household caregiving is driven almost entirely by those with prior caregiving experience. For this group, the RRFW expansion increases the probability of providing within‐household care by 5.7 percentage points. No comparable effect is observed among men who were non‐carers before the reform. Impacts on mental health are positive but again insignificant.

**TABLE 5:** Heterogeneity by pre‐reform caregiving status.

Variables	Provide care				Mental well‐being	
	Within household	Outside household	> 20 hours	> 50 hours	SF‐12	GHQ
	(1)	(2)	(3)	(4)	(5)	(6)
Panel A: Carers						
Men	0.057***	−0.005	0.020	0.001	0.096	0.176
	(0.022)	(0.028)	(0.014)	(0.011)	(0.490)	(0.277)
	3777	4237	3928	3928	4037	4002
Women	−0.003	−0.009	−0.003	−0.012	0.030	−0.156
	(0.022)	(0.037)	(0.019)	(0.009)	(0.594)	(0.346)
	3264	3685	3461	3461	3504	3498
Panel B: Non‐carers						
Men	0.006	−0.003	−0.005	−0.001	0.430	0.047
	(0.006)	(0.007)	(0.003)	(0.001)	(0.286)	(0.152)
	11,835	11,987	11,836	11,836	11,400	11,341
Women	−0.013*	−0.012	−0.006	−0.002	0.012	0.312
	(0.008)	(0.013)	(0.005)	(0.002)	(0.424)	(0.243)
	6546	6669	6554	6554	6350	6334

*Note:* The Table presents two‐way fixed effects estimates of the impact of the expansion of the Right to Request Flexible Work regulation on informal care provision and mental health by the pre‐reform caregiving status. The data are from Waves 1 to Wave 9 of the UK Household Longitudinal Study. The upper panel reports results for the individuals who reported providing any informal care, while the lower panels provide results for those who did not. All specifications control for age and age squared, marital status, education, National Statistics Socio‐Economic Classification, Standard Industry Classification, number of parents in the household, co‐residence with a spouse, number of children, general health, full‐time employment status, and employer size. Individual and time fixed effects are included. Each cell reports the coefficient, standard error (in parentheses), and number of observations (third row).

^*^ *p* < 0.1.

**p < 0.05.

^***^ *p* < 0.01.

For women, the estimates suggest a different result. Women who were non‐carers in the pre‐reform period experience a small decline in the likelihood of providing within‐household care relative to their matched controls. Other changes in caregiving are small and insignificant. As in the main analysis, we do not find statistically significant effects on mental health for either carers or non‐carers. It indicates that the caregiving response to the RRFW expansion is concentrated among men with existing caregiving ties. This is consistent with the results for potential caregivers and supports the interpretation that flexible working mainly facilitates the continuation or reallocation of caregiving among those already connected to caregiving networks, rather than inducing widespread entry into caregiving.

To explore whether the impacts differ across institutional contexts, Table 6 presents estimates separately for Scotland and for the rest of the UK, which is motivated by differences in the social care system. Scotland provides more extensive publicly funded social care (Wang and Costa‐Font 2026) and is likely to reduce baseline reliance on informal care. The results suggest that the caregiving responses to increased access to flexible working are weaker in Scotland than in the rest of the UK. In particular, the increase in within‐household caregiving among men observed in the pooled estimates appears to be concentrated outside Scotland, while estimates for Scotland are smaller and less precisely estimated. The estimates are consistent with a limited role of marginal improvements in workplace flexibility when formal care provision is more generous. In such settings, care needs may already be partially met through public services, reducing the reliance on employment‐based adjustments to absorb care demands. Interestingly, unlike the main results, we observe an improvement in mental health among the Scottish sample with both mental health measures, for both men and women. They may suggest that flexible working delivers psychological benefits in contexts where it does not need to address the conflict between work and care, and hence does not need to be translated into additional caregiving supply.

**TABLE 6:** Heterogeneity by region: Scotland versus rest of the UK.

Variables	Provide care				Mental health	
	Within household	Outside household	> 20 hours	> 50 hours	SF‐12	GHQ
	(1)	(2)	(3)	(4)	(5)	(6)
Scotland						
Men	0.002	−0.035	−0.014	−0.008	1.487**	0.785*
	(0.015)	(0.034)	(0.016)	(0.006)	(0.716)	(0.403)
	1527	1805	1551	1551	1757	1747
Women	−0.019	−0.004	0.000	−0.014	2.051*	0.707
	(0.026)	(0.052)	(0.020)	(0.009)	(1.052)	(0.547)
	1025	1160	1042	1042	1129	1130
Rest of the UK						
Men	0.015**	0.000	0.003	−0.001	0.146	0.043
	(0.007)	(0.009)	(0.004)	(0.003)	(0.235)	(0.128)
	15,995	18,054	16,175	16,175	17,111	17,000
Women	−0.006	−0.004	−0.003	−0.003	−0.181	0.109
	(0.010)	(0.015)	(0.008)	(0.003)	(0.347)	(0.198)
	9355	10,562	9560	9560	10,028	10,003

*Note:* The Table reports two‐way fixed effects estimates of the impact of the expansion of the Right to Request Flexible Work regulation on informal care provision and mental health by gender, focusing on subsamples of Scotland and the rest of the UK. The data are from Waves 1–9 of the UK Household Longitudinal Study. All models control for age and age squared, marital status, education, National Statistics Socio‐Economic Classification, Standard Industry Classification, number of parents in the household, co‐residence with a spouse, number of children, general health, full‐time employment status, and employer size. Individual and time fixed effects are included. Robust standard errors are clustered at the individual level.

^*^ *p* < 0.1.

^**^ *p* < 0.05.

***p < 0.01.

In the rest of the UK, where formal care provision is more limited, greater workplace flexibility appears to facilitate increased caregiving involvement, particularly among men who are traditionally less involved in unpaid care and may face stronger work‐care constraints under rigid working arrangements. In this setting, the absence of clear improvements in mental health does not necessarily imply that workplace flexibility has no mental health benefits. Rather, it may indicate that the reform helped offset the negative mental health consequences that would otherwise arise from increased caregiving responsibilities under inflexible working conditions. In other words, workplace flexibility may have enabled individuals to absorb additional caregiving demands without experiencing corresponding deterioration in mental well‐being.

### 4.5 Discussion of Mechanisms

We next investigate the mechanisms underlying the observed effects of the RRFW expansion, following the conceptual framework outlined earlier. Using the baseline DiD specification, we examine whether the reform affected individuals' time allocation or the organisation of their work schedules.

Table 7 reports the results for time use and work schedule variables. Overall, the estimates indicate little evidence of changes in total time allocated to work, overtime, commuting, or housework. Instead, the main effects operate through changes in work schedules and autonomy. For men, the probability of working regular daytime hours declines by 3.8 percentage points, suggesting a shift toward more varied or non‐standard schedules. For both men and women, access to RRFW significantly increases reported autonomy over working hours, with a larger effect for women (6.7 percentage points) than for men (3.6 percentage points).

**TABLE 7:** Estimated impacts of RRFW on mediating variables.

Variables	Time use				Work schedule			
	Work hours	Overtime hours	Commuting hours	Housework hours	Weekend work	Regular daytime work	Working‐hour autonomy	Working‐manner autonomy
	(1)	(2)	(3)	(4)	(5)	(6)	(7)	(8)
Panel A: Men								
Treated × post	0.097	0.068	0.007	0.034	0.002	−0.038**	0.036**	0.003
	(0.154)	(0.196)	(0.011)	(0.189)	(0.017)	(0.015)	(0.018)	(0.014)
Observations	19,876	19,755	19,286	9491	9069	9045	9060	9063
R‐squared	0.740	0.503	0.560	0.603	0.669	0.728	0.614	0.513
Panel B: Women								
Treated × post	−0.020	0.105	−0.001	−0.019	−0.002	−0.011	0.067***	0.026
	(0.198)	(0.191)	(0.011)	(0.333)	(0.021)	(0.020)	(0.024)	(0.020)
Observations	11,725	11,668	11,401	5648	5343	5322	5340	5332
R‐squared	0.860	0.508	0.662	0.651	0.730	0.681	0.584	0.516

*Note:* The Table presents two‐way fixed effects estimates of the impact of the expansion of the Right to Request Flexible Work regulation on mediating variables by gender. The data are from Waves 1 to Wave 9 of the UK Household Longitudinal Study. All specifications control for age and age squared, marital status, education, National Statistics Socio‐Economic Classification, Standard Industry Classification, number of parents in the household, co‐residence with a spouse, number of children, general health, full‐time employment status, and employer size. Individual and time fixed effects are included. Robust standard errors are clustered at the individual level.

*p < 0.1.

^**^ *p* < 0.05.

^***^ *p* < 0.01.

Table 8 examines whether these changes are accompanied by a reallocation of domestic tasks within households. The results reveal that men experience a 3.7 percentage point increase in the likelihood of being mainly responsible for cooking. For women, there is a small decline in main responsibility for gardening. Although these effects are modest, they point toward increased male involvement in domestic tasks and improved intra‐household coordination.

**TABLE 8:** Estimated impacts of RRFW on within‐couple domestic labour.

Variables	Cooking	Grocery shopping	Cleaning	Washing	Gardening
	(1)	(2)	(3)	(4)	(5)
Panel A: Men					
Treated × post	0.037***	0.004	0.007	0.009	0.012
	(0.014)	(0.014)	(0.013)	(0.013)	(0.019)
Observations	7014	7013	7014	7014	6998
R‐squared	0.681	0.670	0.593	0.522	0.693
Panel B: Women					
Treated × post	−0.007	0.006	−0.021	0.008	−0.044*
	(0.025)	(0.025)	(0.025)	(0.024)	(0.023)
Observations	3484	3484	3484	3483	3477
R‐squared	0.741	0.721	0.701	0.693	0.689

*Note:* The Table presents two‐way fixed effects estimates of the impact of the expansion of the Right to Request Flexible Work regulation on domestic labour within couples. The data are from Waves 1 to Wave 9 of the UK Household Longitudinal Study. All specifications control for age and age squared, marital status, education, National Statistics Socio‐Economic Classification, Standard Industry Classification, number of parents in the household, co‐residence with a spouse, number of children, general health, full‐time employment status, and employer size. Individual and time fixed effects are included. Robust standard errors are clustered at the individual level.

^*^ *p* < 0.1.

**p < 0.05.

^***^ *p* < 0.01.

## 5 Robustness and Placebo Analyses

Next, we perform a series of robustness checks to evaluate the stability of the baseline DiD estimates. Table 9 presents results using alternative matching procedures instead of the baseline PSM approach. Specifically, we employ entropy balancing, k‐nearest neighbour matching with k equal to 3, radius matching, and kernel matching. Although these methods differ in how control observations are selected or weighted, each is designed to improve covariate balance between treatment and control groups. Across all specifications, the main results remain robust. We consistently find an increase in within‐household caregiving among men following the RRFW expansion, and some specifications also indicate a higher likelihood of men providing more than 20 hours of care per week. For women, consistent with the baseline analysis, we find no statistically significant effects on caregiving behaviour or mental health outcomes.

**TABLE 9:** Robustness checks using alternative PSM specifications.

Variables	Provide care				Mental health	
	Within household	Outside household	> 20 hours	> 50 hours	SF‐12	GHQ
	(1)	(2)	(3)	(4)	(5)	(6)
Entropy balancing						
Men	0.010**	0.002	0.008***	0.000	0.144	0.048
	(0.005)	(0.007)	(0.003)	(0.002)	(0.171)	(0.092)
	35,299	39,668	35,701	35,701	37,843	37,552
Women	−0.003	0.003	−0.003	−0.001	−0.080	0.105
	(0.006)	(0.011)	(0.005)	(0.002)	(0.249)	(0.136)
	44,539	49,062	45,228	45,228	46,952	46,735
k‐Nearest neighbour matching (*k* = 3)						
Men	0.010*	0.002	0.005*	−0.000	0.076	0.025
	(0.005)	(0.007)	(0.003)	(0.002)	(0.178)	(0.100)
	25,032	28,319	25,354	25,354	26,965	26,788
Women	0.000	0.005	−0.003	−0.001	−0.062	0.085
	(0.007)	(0.012)	(0.005)	(0.003)	(0.271)	(0.149)
	18,454	20,665	18,799	18,799	19,741	19,685
Radius matching						
Men	0.009**	0.001	0.007**	0.001	0.090	0.054
	(0.005)	(0.006)	(0.003)	(0.002)	(0.161)	(0.089)
	35,284	39,637	35,686	35,686	37,822	37,530
Women	−0.002	0.003	−0.003	0.000	−0.003	0.122
	(0.006)	(0.011)	(0.005)	(0.002)	(0.247)	(0.135)
	44,531	49,054	45,220	45,220	46,944	46,727
Kernel matching						
Men	0.009**	0.002	0.007**	0.001	0.091	0.052
	(0.005)	(0.006)	(0.003)	(0.002)	(0.161)	(0.089)
	35,291	39,660	35,693	35,693	37,839	37,548
Women	−0.002	0.003	−0.003	0.000	−0.003	0.122
	(0.006)	(0.011)	(0.005)	(0.002)	(0.247)	(0.135)
	44,531	49,054	45,220	45,220	46,944	46,727

*Note:* The Table reports two‐way fixed effects estimates of the impact of the expansion of the Right to Request Flexible Work regulation on informal care provision and mental health by gender, using alternative PSM specifications. Panels correspond to different matching methods: entropy matching, nearest neighbour matching with *k* = 3, radius matching, and kernel matching. The data are from Waves 1–9 of the UK Household Longitudinal Study. All models control for age and age squared, marital status, education, National Statistics Socio‐Economic Classification, Standard Industry Classification, number of parents in the household, co‐residence with a spouse, number of children, general health, full‐time employment status, and employer size. Individual and time fixed effects are included. Robust standard errors are clustered at the individual level.

^*^ *p* < 0.1.

^**^ *p* < 0.05.

^***^ *p* < 0.01.

We further examine a range of mental health outcomes using dichotomous indicators, with the results reported in Table 10. In addition to the main continuous measures of mental health, we recode both variables into binary indicators distinguishing relatively poorer from better mental health states. For the SF‐12 MCS, we apply a threshold of 42, consistent with the literature identifying scores below this level as indicative of poorer mental health functioning (Ware et al. 1995; Kim et al. 2024). For the reversed GHQ Likert score, we use a cutoff of 21^7^ to capture lower levels of recent psychological distress (Ponizovsky et al. 2018). For both measures, the binary indicator takes the value of one if the individual is classified as being in a better mental health state, and zero otherwise.

**TABLE 10:** Robustness checks using dichotomous mental health outcomes.

Variables	Dichotomous SF‐12	Dichotomous GHQ
	(1)	(2)
Men	0.013	0.020**
	(0.010)	(0.009)
	18,884	18,762
Women	0.002	0.016
	(0.015)	(0.014)
	11,157	11,133

*Note:* The Table reports two‐way fixed effects estimates of the impact of the expansion of the Right to Request Flexible Work regulation on dichotomous mental health indicators by gender. The data are from Waves 1–9 of the UK Household Longitudinal Study. All models control for age and age squared, marital status, education, National Statistics Socio‐Economic Classification, Standard Industry Classification, number of parents in the household, co‐residence with a spouse, number of children, general health, full‐time employment status, and employer size. Individual and time fixed effects are included. Robust standard errors are clustered at the individual level.

*p < 0.1.

^**^ *p* < 0.05.

***p < 0.01.

The results indicate a statistically significant increase in the probability of reporting better GHQ mental health outcomes among men following the expansion of workplace flexibility. These findings complement the results based on the continuous mental health measures and provide further evidence that flexible working arrangements may help reduce short‐term psychological distress among men. The stronger effects observed under the binary specification may suggest that the reform primarily influenced individuals near clinically or psychologically relevant thresholds of distress, rather than producing substantial shifts across the entire distribution of mental health scores. Workplace flexibility may have been particularly important in preventing individuals from falling into relatively poor mental health states, even if average changes in continuous mental health measures remain limited.

Table 11 presents the results of a placebo test designed to further assess the validity of the empirical strategy. We restrict the sample to individuals who already had access to flexible working arrangements prior to the reform and compare those who did not use flexibility before 2014 (placebo treated) with those who did (placebo control). For this group, the RRFW expansion should not generate a discrete change in constraints. Applying the same PSM‐DiD framework yields estimates that are close to zero and statistically insignificant for both caregiving and mental health outcomes among men and women. These findings suggest that the empirical design does not mechanically generate treatment effects in the absence of a policy‐induced change.

**TABLE 11:** Placebo test.

Variables	Provide care				Mental health	
	Within household	Outside household	> 20 hours	> 50 hours	SF‐12	GHQ
	(1)	(2)	(3)	(4)	(5)	(6)
Men	−0.000	0.009	0.005	0.002	0.144	0.045
	(0.006)	(0.008)	(0.004)	(0.002)	(0.201)	(0.112)
	30,558	34,556	30,923	30,923	32,997	32,767
Women	0.006	−0.004	−0.002	−0.001	−0.285	−0.042
	(0.005)	(0.009)	(0.005)	(0.002)	(0.196)	(0.111)
	28,540	32,021	29,099	29,099	30,583	30,449

*Note:* The Table reports results from a placebo test which compares the alternative “treated” group consisting of employees who have access to flexible working options but do not use them, with the alternative “control” group that includes employees with access who do use them. The data are from Waves 1–9 of the UK Household Longitudinal Study. All models control for age and age squared, marital status, education, National Statistics Socio‐Economic Classification, Standard Industry Classification, number of parents in the household, co‐residence with a spouse, number of children, general health, full‐time employment status, and employer size. Individual and time fixed effects are included. Each cell reports the coefficient, standard error (in parentheses), and number of observations (third row).

*p < 0.1.

**p < 0.05.

***p < 0.01.

## 6 Discussion and Conclusion

This study examines how enhancing job flexibility shapes informal caregiving and mental health, exploiting the 2014 expansion of flexible working arrangements (FWAs) in the UK. Using longitudinal data from the UK Household Longitudinal Study within an event study and Difference‐in‐Differences framework combined with propensity score matching, we provide evidence that expanded access to FWAs through the RRFW reform generated clearly gender‐differentiated responses in caregiving.

We find that the FWAs reform exerted different effects across genders. More specifically, the results show that FWAs led to a sustained increase in the likelihood of providing informal care within the household among men, which appears to operate primarily through changes in work schedules rather than substantial reductions in total working time. In particular, men experienced a decline in regular daytime work and greater autonomy over working hours. It is likely that these changes relaxed binding time and coordination constraints that previously limited men's ability to participate in caregiving. The findings therefore highlight the importance of temporal flexibility in enabling men to take on a more active caregiving role, facilitating a reallocation of care within households without requiring exits from employment or large reductions in paid work hours.

Among potential female caregivers, the main response appears at the intensive margin of care provision. Increased control over working hours following the RRFW expansion coincided with a reduction in very high‐intensity caregiving, particularly care exceeding 50 hours per week. Rather than increasing overall caregiving, flexibility reduces extreme care burdens among them. The estimates are consistent with a partial rebalancing of responsibilities within households, whereby greater male involvement allows women to reduce the most demanding forms of care while remaining attached to paid employment.

We also find some evidence of positive effects on mental health, particularly among men. This suggests that increased involvement in family and caregiving activities, when combined with greater control over working time, does not necessarily reduce well‐being and may even improve it through lower work‐family conflict and improved household coordination. By contrast, women already carried substantially higher caregiving responsibilities prior to the reform, implying smaller marginal gains in well‐being. Although many estimates are statistically insignificant, the overall pattern suggests that workplace flexibility may have helped offset potential mental health deterioration associated with informal caregiving. Evidence from Scotland further supports this interpretation, as weaker reliance on informal care coincides with clearer mental health improvements. At the same time, the limited overall effects may reflect the dual role of flexibility. While reducing work‐care conflict, flexibility may also increase total role demands by enabling additional caregiving responsibilities.

Our estimates align with a growing literature showing that workplace flexibility can reduce gender disparities in unpaid work by encouraging greater male involvement in caregiving and domestic tasks (Lyttelton et al. 2022; Patnaik 2019). Prior research highlights that rigid working arrangements disproportionately constrain men's participation in caregiving, even when preferences exist, whereas flexible arrangements can relax these constraints and shift household behaviour in ways that narrow gender gaps (Leshchenko and Chung 2025; Inoue et al. 2024). This paper also contributes to the RRFW literature by focusing on informal caregiving for adults with disabilities or long‐term health conditions. In addition, it extends existing work by examining caregiving intensity and mental health within an event study framework that allows for transparent assessment of pre‐trends. In addition to prior studies such as Xue et al. (2026), Spearing (2025), and Agnoletto (2024), the findings point to new insights on mental health effects that depend on job flexibility, caregiving intensity and time reallocation within households.

Despite the strengths of the empirical strategy, several limitations remain. The estimated effects rely on observed characteristics used in matching and regression adjustment, meaning that unobserved workplace heterogeneity cannot be fully ruled out. Differences in job quality, managerial practices, career expectations, or workplace culture may still confound the estimates. While the event study shows no evidence of differential pre‐trends, it cannot fully rule out time‐varying unobservables, such as industry shocks or broader shifts toward flexible working that coincide with the reform. In addition, selection into caregiving prior to the reform may still bias estimates if individuals with greater care responsibilities systematically sorted into more flexible jobs.

Measurement issues also represent an important limitation. Caregiving outcomes rely on self‐reported survey data, which may be subject to recall and reporting bias and may vary across individuals and genders. Similarly, flexible working arrangements are measured through self‐reported access and use rather than objective employer‐level measures, limiting precision. Although robustness checks support the main findings, these limitations imply that some uncertainty remains regarding the exact magnitude of the estimated effects.

The findings carry important policy implications. The expansion of RRFW suggests that improving access to workplace flexibility can increase caregiving participation, particularly among men, without harming mental health. By easing time and coordination constraints, flexible working arrangements can enable workers to take on greater caregiving responsibilities while remaining in employment. The results also indicate potential reductions in gender disparities in informal caregiving, with increased male involvement and reduced extreme caregiving burdens among women, achieved without direct financial incentives or wage reductions. More broadly, the effectiveness of flexibility policies appears to depend on the institutional context of formal social care provision, with stronger well‐being effects where public care systems reduce reliance on informal care.

Several directions for future research emerge. First, richer and more objective measures of workplace flexibility, ideally linked to employer or administrative data, would improve identification. Second, more detailed couple‐level and household‐level analyses could shed light on intra‐household coordination and bargaining. Examining how both partners' access to flexibility jointly shapes caregiving, labour supply, and mental health would further deepen understanding of the mechanisms through which workplace flexibility reshapes household behaviour.

## Conflicts of Interest

The authors declare no conflicts of interest.

## Data Availability

The data that support the findings of this study are available from Institute for Social and Economic Research (ISER). Restrictions apply to the availability of these data, which were used under license for this study. Data are available from http://doi.org/10.5255/UKDA‐SN‐6614‐21 with the permission of Institute for Social and Economic Research (ISER).

## Endnotes

^1^
In this paper, “caregivers” refers to individuals providing care for adults with disabilities or health conditions and excludes childcare.

^2^
In this paper, potential caregivers are defined as employees whose family members have disabilities or long‐term health conditions requiring care.

^3^
The application process requires employees to submit a written request specifying the desired changes to their working arrangements. Employers must consider and respond to these requests within a reasonable time frame and may refuse them only on recognised business grounds. When approved, changes typically become permanent unless a temporary arrangement is explicitly agreed.

^4^
The right was further amended by the Employment Relations (Flexible Working) Act 2023, which, from April 2024, made the right to request flexible working a “day one” right.

^5^
UKHLS defines family members broadly, including partners, parents, parents‐in‐law, grandparents, aunts, uncles, and other extended relatives.

^6^
Differences in sample size across flexibility variables arise because information on flexible working was collected only in even‐numbered survey waves. See Table A2 for details.

^7^
This threshold is based on the reversed GHQ Likert score. It corresponds to the conventional cutoff of 15 on the original GHQ Likert scale, where higher values indicate greater psychological distress.

## Appendix

**TABLE A1:** Balance of covariates for unmatched and matched samples (baseline wave).

Characteristics	Unmatched sample			Matched sample		
	Control	Treated	*p*‐value	Control	Treated	*p*‐value
Age	41.347	42.153	0.001***	41.907	42.156	0.424
Male	0.428	0.663	0.000***	0.625	0.662	0.005***
Married	0.745	0.743	0.758	0.737	0.742	0.644
Education: Degree	0.346	0.217	0.000***	0.248	0.218	0.011**
Education: Other higher education	0.140	0.128	0.077*	0.140	0.127	0.190
Education: A level etc	0.218	0.255	0.000***	0.238	0.255	0.172
Education: GCSE etc	0.204	0.250	0.000***	0.249	0.251	0.904
Education: Other qualification	0.058	0.095	0.000***	0.081	0.095	0.09*
Education: No qualification	0.035	0.056	0.000***	0.044	0.055	0.073*
NS‐SEC: Higher management	0.061	0.049	0.013**	0.049	0.049	0.977
NS‐SEC: Higher professional	0.094	0.057	0.000***	0.065	0.057	0.241
NS‐SEC: Lower management & professional	0.346	0.283	0.000***	0.304	0.284	0.109
NS‐SEC: Intermediate	0.170	0.128	0.000***	0.148	0.129	0.043**
NS‐SEC: Lower supervisory & technical	0.002	0.001	0.021**	0.132	0.157	0.012**
NS‐SEC: Semi‐routine	0.077	0.159	0.000***	0.169	0.171	0.814
NS‐SEC: Routine	0.173	0.172	0.837	0.134	0.154	0.042**
SIC: Agriculture, forestry and fishing	0.003	0.009	0.000***	0.006	0.008	0.422
SIC: Mining and quarrying	0.004	0.007	0.005***	0.007	0.007	0.119
SIC: Manufacturing	0.093	0.204	0.000***	0.183	0.204	0.068*
SIC: Electricity, gas, steam and air conditioning supply	0.008	0.011	0.122	0.012	0.011	0.818
SIC: Water supply; sewerage, waste management activities	0.006	0.015	0.000***	0.011	0.015	0.201
SIC: Construction	0.029	0.078	0.000***	0.058	0.079	0.005***
SIC: Wholesale and retail trade	0.129	0.145	0.019**	0.142	0.144	0.812
SIC: Accommodation and food service activities	0.044	0.072	0.000***	0.063	0.072	0.206
SIC: Transport and storage	0.031	0.037	0.940	0.039	0.037	0.708
SIC: Information and communication	0.038	0.029	0.013**	0.032	0.028	0.457
SIC: Financial and insurance activities	0.045	0.028	0.000***	0.034	0.028	0.252
SIC: Real estate activities	0.009	0.012	0.139	0.015	0.012	0.525
SIC: Professional, scientific and technical activities	0.049	0.040	0.045**	0.044	0.040	0.511
SIC: Administrative and support service activities	0.037	0.047	0.019**	0.051	0.047	0.593
SIC: Public administration and defense	0.108	0.044	0.000***	0.054	0.043	0.057**
SIC: Education	0.132	0.075	0.000***	0.084	0.075	0.322
SIC: Human health and social work activities	0.200	0.115	0.000***	0.132	0.115	0.072*
SIC: Arts, entertainment and recreation	0.018	0.016	0.328	0.015	0.016	0.735
SIC: Other service activities	0.017	0.015	0.340	0.018	0.015	0.330
SIC: Activities of households as employers	0.001	0.002	0.006***	0.002	0.002	0.813
SIC: Activities of extraterritorial organisations	0.001	0.002	0.121	0.001	0.002	0.483

*Note:* The Table presents covariate balance tests for treated and control groups before and after propensity score matching. The sample includes employees from Wave 4 (2012/2013) of the UK Household Longitudinal Study. Variables include demographic characteristics (age, gender, marital status, and education), National Statistics Socio‐Economic Classification categories, and Standard Industry Classification codes.

^*^ *p* < 0.1.

^**^ *p* < 0.05.

^***^ *p* < 0.01.

**TABLE A2:** Definitions and survey frequency of key variables.

Variable	Definition	Survey frequency
Work arrangements		
Number of FWAs	The number of flexible work arrangements available at the workplace	Every 2 years (even waves)
Use any FWAs	Whether use any of the flexible work arrangements (yes = 1, no = 0)	Every 2 years (even waves)
Use informal FWAs	Whether able to vary working hours on an informal basis (yes = 1, no = 0)	Every 2 years (even waves)
Care arrangements		
Care within household	Whether provide informal care within the household (yes = 1, no = 0)	Every year
Care outside household	Whether provide informal care outside the household (yes = 1, no = 0)	Every year
Care > 20 hours	Whether spend 20 and over hours on caring per week (yes = 1, no = 0)	Every year
Care > 50 hours	Whether spend 50 and over hours on caring per week (yes = 1, no = 0)	Every year
Mental health		
SF‐12 MCS	SF‐12 mental component summary: Mental functioning score (scale 0–100)	Every year
GHQ likert score	General health questionnaire: Amount of distress (scale 0–36)	Every year
Mediators		
Work hours	Number of hours normally worked per week	Every year
Overtime work hours	Number of overtime hours in a normal week	Every year
Commuting hours	Hours spent traveling to work	Every year
Housework hours	Hours spent per week on housework	Every 2 years (even waves)
Weekend work	Usually worked on weekends (yes = 1, no = 0)	Every 2 years (even waves)
Regular daytime work	Usually worked during the day (yes = 1, no = 0)	Every 2 years (even waves)
Working‐hour autonomy	Autonomy over time you start or finish your working day (Some/A lot = 1, None/A little = 0)	Every 2 years (even waves)
Working‐manner autonomy	Autonomy over how you do your work (Some/A lot = 1, None/A little = 0)	Every 2 years (even waves)

*Note:* The Table provides definitions and survey frequencies for key variables used in the analysis. Caregiving intensity is measured based on reported weekly caregiving hours in bands: 0–4, 5–9, 10–19, 20–34, 35–49, 50–99, and 100 or more hours. Variables measuring autonomy over work hours and work manner are transformed into binary indicators from original four‐category responses (None, A little, Some, A lot). Survey frequencies indicate how often each variable is collected in the UK Household Longitudinal Study.

**TABLE A3:** Dynamic effects of RRFW on informal care provision across waves.

Year	Men				Women			
	Care within household	Care outside household	Care > 20 hrs	Care > 50 hrs	Care within household	Care outside household	Care > 20 hrs	Care > 50 hrs
	(1)	(2)	(3)	(4)	(5)	(6)	(7)	(8)
2009/10	0.010	0.012	−0.011	0.002	0.016	0.039	0.000	0.009
	(0.012)	(0.018)	(0.009)	(0.006)	(0.013)	(0.026)	(0.013)	(0.007)
2010/11	0.003	0.009	−0.002	0.003	0.012	0.018	−0.008	−0.005
	(0.009)	(0.014)	(0.007)	(0.004)	(0.011)	(0.021)	(0.011)	(0.006)
2011/12	0.012	−0.001	0.011*	0.004	0.009	−0.005	−0.012	0.004
	(0.008)	(0.014)	(0.006)	(0.004)	(0.010)	(0.019)	(0.011)	(0.006)
Reference Year								
2013/14	0.010	0.004	0.006	0.002	0.006	−0.003	0.003	−0.004
	(0.008)	(0.013)	(0.007)	(0.004)	(0.010)	(0.019)	(0.011)	(0.006)
2014/15	0.030***	−0.005	−0.003	−0.001	0.003	0.003	−0.022	−0.011*
	(0.011)	(0.016)	(0.008)	(0.005)	(0.014)	(0.025)	(0.013)	(0.006)
2015/16	0.028**	0.000	0.004	0.007	0.003	−0.003	−0.016	0.000
	(0.011)	(0.015)	(0.007)	(0.005)	(0.015)	(0.025)	(0.013)	(0.006)
2016/17	0.031**	0.019	−0.003	−0.003	−0.007	−0.015	−0.010	0.000
	(0.012)	(0.018)	(0.008)	(0.006)	(0.017)	(0.027)	(0.015)	(0.007)
2017/18	0.018	−0.012	0.007	0.001	−0.011	0.019	−0.001	0.005
	(0.012)	(0.019)	(0.007)	(0.005)	(0.016)	(0.029)	(0.014)	(0.008)

*Note:* The Table reports event study estimates of the impact of RRFW on informal care provision by gender, using Equation (1). The analysis uses data from Waves 1 to Wave 9 of the UK Household Longitudinal Study, with Wave 4 (2012/2013) as the baseline.

^*^ *p* < 0.1.

^**^ *p* < 0.05.

^***^ *p* < 0.01.
